# Methodology for Digital Transformation with Internet of Things and Cloud Computing: A Practical Guideline for Innovation in Small- and Medium-Sized Enterprises

**DOI:** 10.3390/s21165355

**Published:** 2021-08-09

**Authors:** Yu Liu, Zhongjun Ni, Magnus Karlsson, Shaofang Gong

**Affiliations:** Department of Science and Technology, Campus Norrköping, Linköping University, 60221 Norrköping, Sweden; yu.a.liu@liu.se (Y.L.); zhongjun.ni@liu.se (Z.N.); magnus.b.karlsson@liu.se (M.K.)

**Keywords:** digital transformation, Internet of Things, cloud computing, vertical plant wall

## Abstract

Researches on the Internet of Things (IoT) and cloud computing have been pervasive in both the academic and industrial world. IoT and cloud computing are seen as cornerstones to digital transformation in the industry. However, restricted by limited resources and the lack of expertise in information and communication technologies, small- and medium-sized enterprises (SMEs) have difficulty in achieving digitalization of their business. In this paper, we propose a reference framework for SMEs to follow as a guideline in the journey of digital transformation. The framework features a three-stage procedure that covers business, technology, and innovation, which can be iterated to drive product and business development. A case study about digital transformation taking place in the vertical plant wall industry is detailed. Furthermore, some solution design principles that are concluded from real industrial practice are presented. This paper reviews the digital transformation practice in the vertical plant wall industry and aims to accelerate the pace of SMEs in the journey of digital transformation.

## 1. Introduction

Digital transformation has been witnessed to take place across all industries and business sectors, though at different paces. Digital transformation aims to leverage digital technologies to enhance products and services, optimize operation efficiency, automate industrial processes, improve customer experiences, and transform business models [1]. The road map of digital transformation is in line with the emergence of new digital technologies, among which two representatives that have profound influences on the industry are Internet of Things (IoT) and cloud computing.

**Figure 1:**
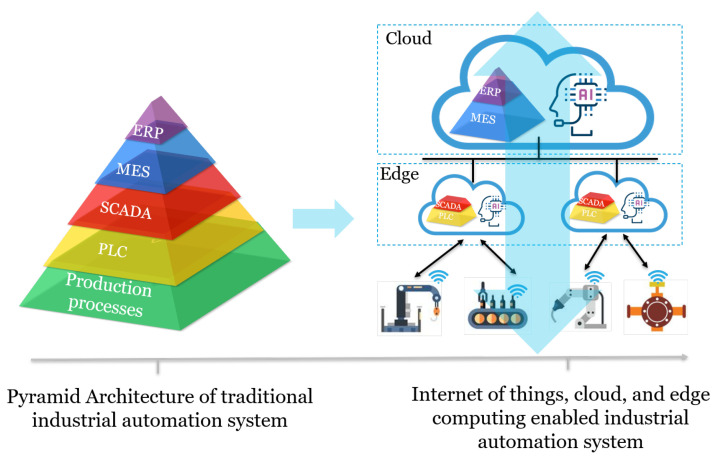
An illustration of the digital transformation in industrial automation system. The traditional industrial automation system adopts a pyramid architecture where the production process, programmable logic controller (PLC), supervisory control and data acquisition (SCADA), manufacturing execution system (MES), and enterprise resource planning (ERP) layers are strictly hierarchical. By leveraging IoT and cloud computing, the connectivity and computation capabilities are enhanced, and artificial intelligence derived from data is distributed across the full stack of the system.

Take the IoT and cloud-enabled digital transformation in industrial automation system as an example. As shown in Figure 1, the architecture of a traditional industrial automation system is highly hierarchical, which is a layered structure including the production process layer, programmable logic controller (PLC) layer, supervisory control and data acquisition (SCADA) layer, manufacturing execution system (MES) layer, and enterprise resource planning (ERP) layer following a bottom-up order [2], in which machine-to-machine (M2M) communications are utilized. In such a system, functionalities of each layer are predefined. The boundaries between each layer are clear, which greatly restrict interactions across the full stack. In recent years, the Industry 4.0 paradigm has been evolving towards Industry 5.0, benefiting from which, industry IoT (IIoT) is introduced and dedicated to elevating the connectivity of the industrial automation system to a higher dimension. With massively deployed sensors and actuators on the production floor, IoT enables the stakeholder to monitor the industrial process in real-time. Backed by cloud computing, which features superior computation and storage capabilities and a widespread distribution, a large volume of IIoT data can be processed and analyzed. Thus, intelligent functions such as anomaly detection [3] and predictive maintenance [4] that are derived from insightful data analytics can be created. Incorporating the evolving cloud computing architecture brings enhanced processing capability and improved scalability. Therefore, digital transformation drives a new business value proposition for today’s industrial automation system.

In light of the benefits, strategies to perform digital transformation in the industry and its impact have been studied by the academic and industrial community [5,6,7,8,9] as well as the challenges [10,11,12] that hinder business stakeholders from digitalization. However, as pointed out by [13], digital transformation is easier to articulate than to execute. This is a crucial issue encountered by small- and medium-sized enterprises (SMEs) in the manufacturing industry. On the one hand, lack of knowledge in information and communication technology (ICT) and limited budget and resources imply tremendous difficulties for SMEs. On the other hand, falling behind in the digital competition can lead to worse market performance. Therefore, an applicable framework that grants SMEs the capability to bridge the gap is under demand, which forms the fundamental motivation of this study.

Specifically, this paper limits the scope to SMEs within the manufacturing industry that envisions digital transformation to reshape the product and service while IoT and cloud computing are the key enablers. It provides an easy-to-follow framework for practitioners to think of and implement digital solutions while being constrained by a lack of relevant experience, expertise, and competence in the ICT domain. Based on the framework, an experience of driving digital transformation in the vertical plant wall industry is shared and exemplified together with instructive solution design principles. This paper originates from a real collaborative research project between Linköping University and Vertical Plant Systems AB, and is related to a series of earlier publications and experiences accumulated from the project. The proposed methodology cannot only be utilized as an accelerator for research and development (R&D) for enterprises running businesses in a similar sector, but also referenced by industries that encounter obstacles when adopting IoT and cloud-enabled digital transformation.

Major contributions of the study are as follows:

- Proposed an applicable framework for SMEs in traditional manufacturing industry to drive IoT and cloud-computing-enabled digital transformation;
- Exemplified the framework with a real case study in the vertical plant wall industry in a detailed approach;
- Highlighted instructive principles for solution design to further accelerate the digitalization process and reduce risk of failure.

The rest of the paper is organized as follows: Section 2 reviews existing digital transformation theories and practices in the literature. Section 3 presents the proposed reference framework for digital transformation in SMEs. Section 4 details the digital transformation process in the vertical plant wall industry. Section 5 presents several instructive principles for IoT solution development that are extracted based on a real industrial digital transformation practice and Section 6 discusses achievements, limitations, and future expectations. Section 7 concludes the paper.

## 2. Related Work

Many researches tend to provide digital transformation strategies on a high point for general enterprises. In [5], a digital road map was built by considering transformations in people, process, technology, and content tracks in order to achieve improved customer experience, organizational efficiency, and integrated information infrastructure. Similarly, in [6], the author highlighted initializing the digital transformation road map by taking consideration of challenges, objectives, capabilities, and actions in turn. The study [14] summarized digital transformation strategies into four categories according to business model readiness and digital technology maturity. In [15], the authors proposed a down-up process model for the development of a digital strategy for manufacturing companies in particular, and emphasized the digital strategy needs to consider functional, business, and corporation levels. The work [16] proposed a method to generate a digital strategy map, taking consideration of corporate profit, customer experience, digital business ecosystem, and digital transformation requirements. References [17,18] presented business process management tools to help SMEs to achieve digital transformation. These work provided insights of digital transformation strategies from a business perspective. However, a detailed analysis or case study about applying the strategy to a real industry is rarely touched.

A series of articles also focus on digital transformations taking place in real industries. For example, the work [13] reviewed 25 companies and concludes customer engagement and digitized solutions as two key strategies for digital transformation in large traditional enterprises. In [11], the authors presented three case studies about how digital transformation is successfully approached and provided a guideline to address the challenges and risks for business managers. In the study [19], the authors showcased an example of digital transformation in the retail industry, which uses IoT to realize inventory control and end up with an information platform. In [20], a case study of how the digital manufacturing ecosystem reshapes the value chain of the food industry was presented, while in [21], the digital transformation experiences of three large enterprises in the electrical and agriculture manufacturing and the telecommunication industries were reviewed. These studies concluded valuable empirical findings of digital transformation in real industry, but a lack of practical guidelines for the technology development path.

As for the IoT and cloud-computing-enabled transformation, the work [8] revealed that the core is to utilize IoT to create smart products and data-driven services, and proposed a four-step reference process for digital transformation, which includes the creation of a strategic business vision, a data model, and solutions as well as decision-making. In [7,9], the authors introduced reference architectures and integration methods to integrate IoT and cloud computing technologies into a digital enterprise architecture. In [12], the authors identified several key challenges to be considered when leveraging IoT in business model development such as data ethics and trust. These works mainly provide a theoretical road map to the digital transformation in the industry without any validation. Plenty of researches in the technical world have also presented detailed architectures and solutions for integrating IoT and cloud computing into the digitalization of different industrial sectors. Some examples about the rail, forest, home appliances, and healthcare industries were presented in [22,23,24,25,26]. However, in these types of studies, technology is highlighted but business-centric thinking is always missing, which can hardly be referenced by SMEs as a methodology.

In this study, we aim to provide a reference framework for SMEs to accomplish digital transformation. Both theoretical and practical perspectives are emphasized. Business thinking and a technology development road map are included to formulate a best practice for business practitioners.

## 3. Digital Transformation Framework

It is the business strategy, not the technology, that drives digital transformation in a company. This has been a common consensus agreed by the industry [27] and is an essential principle for SMEs. Although there are successful stories achieved with the *technology push* approach, it is not feasible and can be risky for SMEs to take this approach, due to the limited resources they can spend and the low fault-tolerance nature of SMEs.

**Figure 2:**
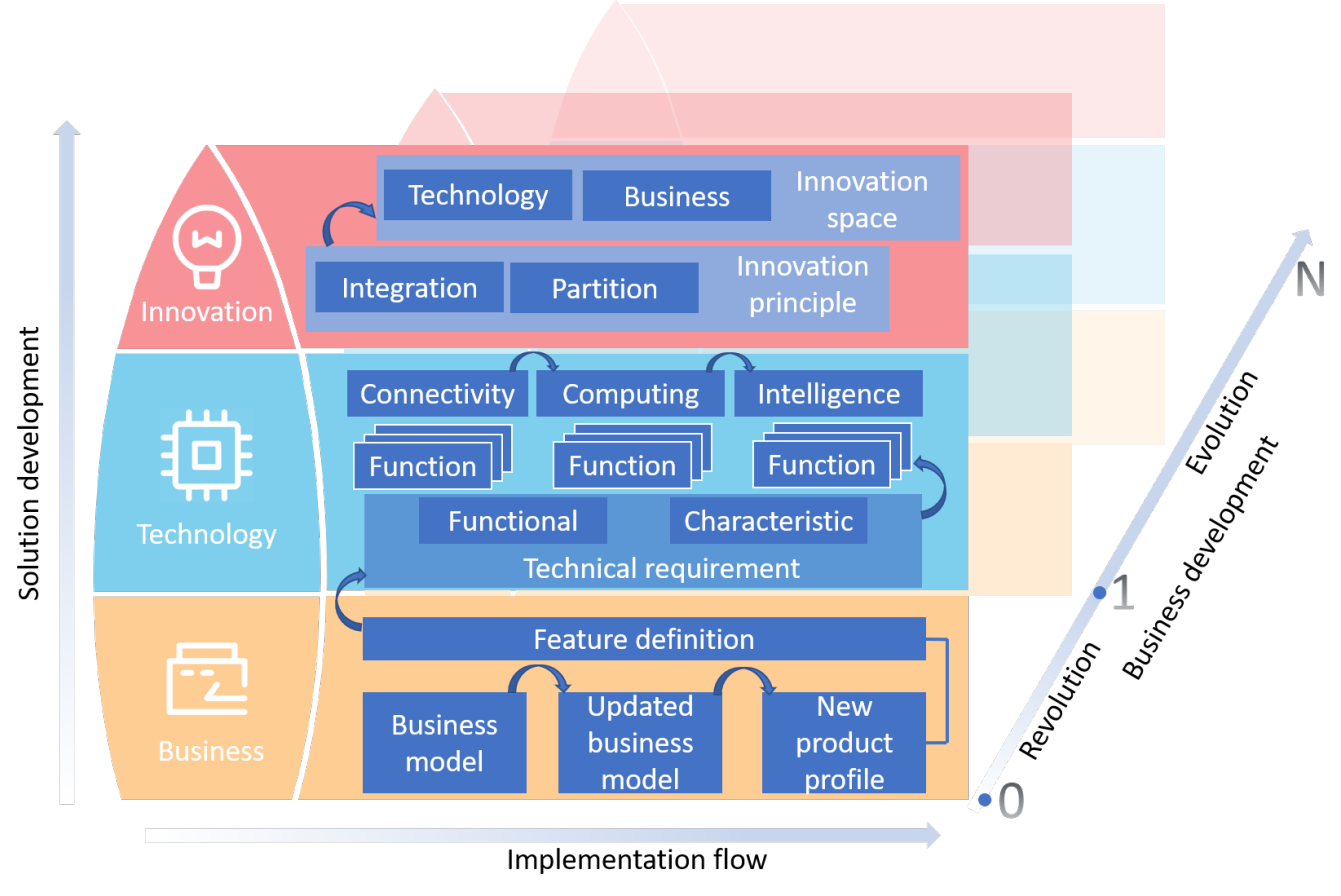
A reference framework for SMEs to accomplish digital transformation with enabling technologies such as IoT and cloud computing. It features a three-stage iteration that covers consideration of business, technology, innovation, and three dimensions of development flows. The framework can be referenced by business stakeholders as a guideline to formulate a technology development road map and to trigger business development.

Figure 2 shows a reference framework aiming for SMEs to implement digital transformation within the manufacturing industry. A three-stage rocket concept is illustrated in the framework to cover considerations of business, technology, and innovation aspects following a bottom-up order. The three-stage architecture signals describe that the digital transformation procedure is business-driven, technology-enabled, and innovation-guided. The framework also highlights three dimensions of development flows, i.e., an implementation flow within each stage, a solution development flow across stages, and a business development flow. The details are explained as follows.

The framework starts the process with a business stage, suggesting that the origin of digital transformation is the business value. Depending on if the goal is to develop a new product or to upgrade an existing product, a business model will be defined or updated to clearly reflect new value creations, customer segments and relations, and revenue streams. This procedure is essential to understand the characteristics of the intended new product (or service), and thus, shall be thoroughly studied. After the business model is defined or refreshed, the key features of the product shall be defined according to the result, which then constitute the fundamentals for the next stage.

The next stage of the framework aims to provide structured thinking to devise the technical solution to accomplish the anticipated goal. First, based on the well-defined features in the business stage, technical requirements are formulated, which include both functional requirements (e.g., monitoring of temperature) and characteristics (e.g., performance metrics such as latency, and properties such as security). The features are verified by these requirements in turn, and each feature will generate several function blocks that are appropriately selected to address the requirements. The final solution is determined by a union of all the function blocks. As a guideline to implement the solution, three phases—namely, connectivity, computing, and intelligence—can be considered in turn. In this step, enabling technologies in the IoT and cloud computing domain can be freely selected to fulfill the technical requirements, such as various wired and wireless communication protocols, application layer messaging protocols, and machine learning and artificial intelligence algorithms, to name a few.

The top stage is where innovations take place. In this stage, two fundamental innovation principles, namely, integration and partition are proposed. The playground to apply innovations can be either technology or business or both. An improvement in technology may bring new business values and trigger business model updates, while a change in business model can incur new technical requirements. In either way, the three-stage procedure shall be revisited and iterated.

In practice, three dimensions of development flows can be followed in the framework. The implementation and solution development flows guide the direction of thinking within and across the three stages, respectively. Iterations of the three-stage procedure will generate the dimension of business development. In this dimension, the first iteration bridges the gap between “0” and “1”, and is seen as a revolution phase while the following iterations constitute the evolution phase. In this way, digital transformation can be accomplished in a step-by-step manner.

## 4. Case Study: The Vertical Plant Wall Industry

In this section, a case study of leveraging the proposed framework to perform digital transformation in the vertical plant wall industry is presented.

### 4.1. The Vertical Plant Wall Industry

**Figure 3:**
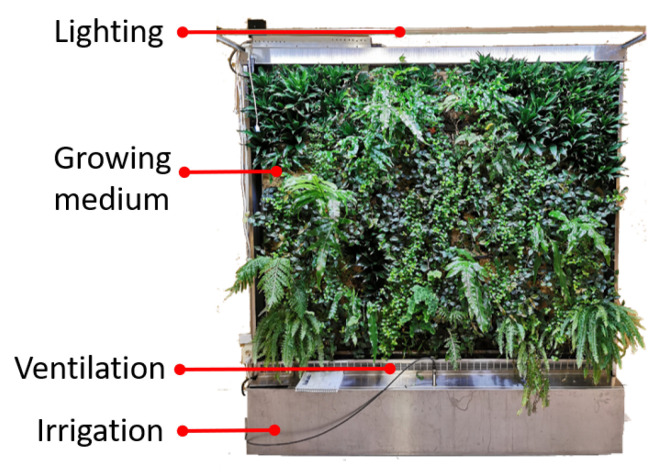
An example of a vertical plant wall system, which integrates lighting, ventilation, irrigation systems, and a growing medium to support green plants to grow on the surface.

As shown in Figure 3, a vertical plant wall (also known as vertical plant system) is a vertical wall with diverse types of green plants growing on the surface. It also has growing medium, irrigation, lighting, and ventilation systems integrated so as to guarantee the plants growing. A plant wall can significantly contribute to indoor environments through evaporation, air purification, and water retention. Researches [28] show that vertical plant walls can effectively purify air pollutants such as particulate matter (PM) and volatile organic compounds (VOC) while stabilizing CO${}_{2}$ concentrations, therefore improving human comfort and work productivity and reducing energy use.

The company Vertical Plant Systems AB (VPS) is a plant wall manufacturer and supplier located in Sweden. According to VPS, existing sales of plant walls are limited to public spaces such as shopping malls, universities, and museums, and the market expansion is moving slowly. This arises from a realistic challenge. Plant walls need regular plant care by staff with relevant expertise, e.g., to fill the water tank, to trim dead or overgrown leaves, and to plant new vegetation, which is quite time-consuming and labor-intensive for a small company with limited staff. Geographically, it restricts VPS to distributing plant walls to remote places as VPS is unable to serve massive customers with the existing "rent out and maintain" business model. Therefore, a digital system that can remotely monitor plant walls and automate plant care is demanded by VPS, which constitutes the motivation for a digital transformation to take place at VPS. A collaborative project between VPS and Linköping University was initiated to accelerate the digitalization and, below, we detail the procedure following the proposed framework.

### 4.2. The Business Stage

**Figure 4:**
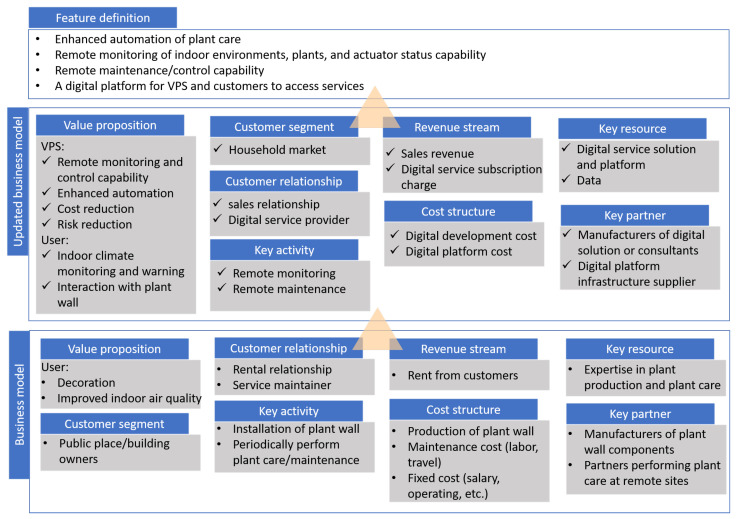
Digital transformation practice for Vertical Plant Systems AB in the business stage. The business model is updated according to the anticipated digital product, and the features of the digital product are defined thereafter.

The first step is to perform business model analysis and recognize what has been updated with the anticipated digital product. There are many tools available for business model analysis, one commonly used is the business model canvas. In Figure 4, the bottom layer shows the existing business model of VPS, in which the valuable proposition, customer segment and relationship, key activities, revenue and cost, and key resources and partner for running the business are identified. Based on the same analysis method, an updated business model is analyzed according to the anticipated digital product, and the key differences are identified.

For instance, in the updated business model shown in Figure 4, VPS expects that a digitalized solution can create new values to the company through remote monitoring and control of plant walls and enhanced automation capability, and to customers through indoor climate monitoring, warning functions, and the possibility to interact with plant walls. The key activities in the business of VPS will switch to remote monitoring and remote maintenance of plant walls instead of regular and frequent on-site maintenance. With the new value proposition, VPS envisions a new customer segment, i.e., the household market to be reached. Accordingly, the customer relationship is enriched from the rental to sale relationship. Besides, VPS also establishes a new relationship with customers by providing digital services through a platform. In this regard, the digital services to the plant wall and the platform together with potentially accumulated data constitute the key resources of VPS. Based on the analysis, four key features of the expected digital solution that are enablers to the business model update are extracted and defined as follows:

- Enhanced automation of plant care;
- Remote monitoring of indoor environments, plants, and actuator status capability;
- Remote maintenance or control capability;
- Digital platform for both VPS and customers to access digital services.

### 4.3. The Technology Stage

The technology stage takes the features defined in the previous step as input. For each feature, a series of technical requirements, i.e., functional and characteristic requirements, are proposed to realize the feature; accordingly, function blocks are created to address the requirements. Each function block contains a technical approach, which can be either a specific software, hardware, or a range of possible selections. The procedure may iterate so that function blocks get refined and clear in each iteration.

#### 4.3.1. Feature 1: Automation

**Figure 5:**
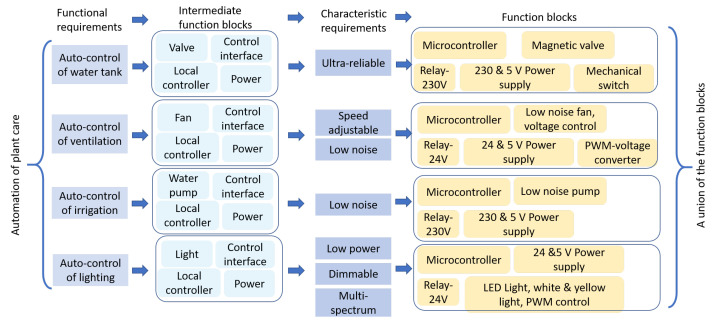
The *automation of plant care* feature extracted from the business stage is taken as input to the technology stage and translated into a union of function blocks that define needed components to achieve the feature.

One example is shown in Figure 5, which illustrates how the enhanced automation of plant care feature is translated into function blocks. To implement the feature, four functional requirements are proposed, i.e., the automatic control of lighting, irrigation, ventilation, and water tank. For each functional requirement, a preliminary combination of function blocks—namely, intermediate function blocks—are generated. For example, to satisfy automatic control of lighting, four intermediate function blocks—namely, light, control interface, local controller, and power—are created. This preliminary block combination is then checked by a series of characteristic requirements that are corresponding to each functional requirement. In the automatic control of lighting case, low power consumption, being dimmable, and multispectrum are defined as characteristic requirements. To meet these requirements, the block of light is refined as an LED light strip with white and yellow dual-color and PWM-control capability, the control interface is refined to relay, the local controller is refined to microcontroller, and the power is refined to a 24 and 5 volt power supply. Taking the same procedure, the other three functional requirements can also generate three groups of function blocks, as shown in the figure. It can be noticed that different combinations of function blocks can share some elements such as the microcontroller. Therefore, the last step is to unite the four groups of function blocks to guarantee that shareable components are identified, and a final solution would cover all the functional and characteristic requirements. After this step, all the components needed to achieve this feature are clearly defined in the final union of function blocks.

#### 4.3.2. Features 2 and 3: Remote Monitoring and Maintenance

**Figure 6:**
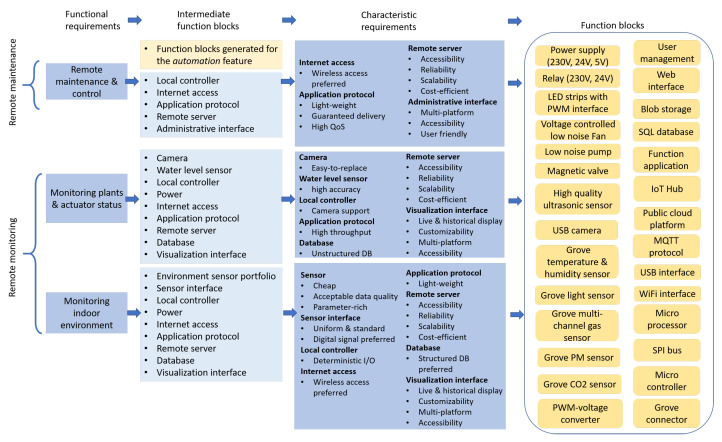
The *remote monitoring* and *remote maintenance* features are verified by functional and characteristic requirements and translated into a union of function blocks.

Figure 6 shows a more complicated example that is to figure out function blocks for the remote monitoring and remote maintenance features. At first, functional requirements corresponding to these two features are proposed, i.e., monitoring of indoor environment, monitoring of plants and actuator status, and remote maintenance and control. To satisfy each of the functional requirements, three preliminary groups of function blocks are created. For instance, to realize indoor environment monitoring, a preliminary function block group that includes environment sensor portfolio, sensor communication, local controller, power, Internet access, application protocol, remote server, database, and visualization interface is worked out, which describes the fundamental components/technologies to achieve the goal. These function blocks are further refined by several characteristic requirements. For example, the sensors portfolio to be used should have low cost with acceptable quality and rich parameter measurement while sensor interfaces should be uniform and digital signals prioritized. These requirements can effectively narrow the range for a selection of sensors. To enable sensor reading, the local controller should fulfill determinism in I/O ports. A wireless approach is preferred for Internet access so as to improve mobility of plant walls. As for the application, considering the lightweight nature of IoT sensor data, a lightweight protocol shall be selected so as to ease the burden of the local controller. Remote server is a vital infrastructure to the solution. A remote server is geographically accessible from a wide range of business locations, able to offer reliable services, can be easily scaled when the business grows, and is cost-efficient for SMEs. The database is required to support structured data of sensor values. The visualization interface needs to support both live and historical records. Additionally, the display methods shall be customizable, and the interface shall be accessible from multiple platforms and any location.

Taking the aforementioned characteristic requirements into account, the intermediate function blocks are refined to a more specific format. A series of cheap environmental sensors with the uniform Grove connector are decided for sensor portfolio. The local controller is refined to a combination of a microcontroller and microprocessor with a WiFi interface. The former guarantees deterministic interfacing to sensors while the latter promises Internet access. A function block of SPI bus is therefore added as a communication channel among them. Considering the demanding requirements for remote server, a public cloud platform is an ideal option to take the role rather than building a private data server.

Based on the same methodology, function blocks corresponding to the other two functional requirements can be generated. By uniting the function blocks together, a final technical solution is worked out. In this step, some function blocks can be further refined. For example, the application protocol is set to message queuing telemetry transport (MQTT) as it has superior performance with respect to lightweight and high throughput, compared with other alternatives such as the hypertext transfer protocol (HTTP) and advanced message queuing protocol (AMQP) [29]. In this regard, an IoT hub service in the public cloud, which is an out-of-box MQTT broker, is selected to manage the communications between the cloud and local field. Function applications that are based on serverless computing are adopted to adapt data into corresponding databases. The visualization interface and administrative interface are also merged to a single web application that is hosted on the cloud in order to meet the requirements.

The union of function blocks figured out in the above two examples already provide a guideline on the technology development road map, including hardware, software, and infrastructure selection. It is also regarded as the technical base for the realization of the last feature—i.e., a *digital service platform*—which aims to leverage the value of data to create smart functions for VPS and customers using machine learning and artificial intelligence, e.g., anomaly detection of indoor climate. This is in accordance with the recommended order of three development phases, i.e., from communication and computing through intelligence that shall be taken into account throughout the whole procedure of solution design. With the union of the function blocks, a final solution for VPS can be confirmed. The implementation details such as connection between components, software development in the local controllers, the architecture in the cloud, and the web interface are presented in this paper [30]. Furthermore, results of intelligent functions to detect anomalies in an indoor environment using machine learning algorithms are detailed in this work [31].

To summarize, from a technical contribution perspective, a complete solution for remote monitoring and management of plant wall systems is proposed and implemented for the plant wall industry for the first time, which supports real-time sensing of indoor climate, autonomous plant maintenance, remote manipulation of actuators, and a centralized interface for visualization and interaction as well as alert functions. Furthermore, machine learning and neuron-network-based anomaly detection methods are developed based on continuously collected sensing data and deployed to the system so as to realize predictive maintenance of plant walls and indoor climate. These features can largely ease the operation cost of the plant wall industry, eliminate the geographic constraints to sale, and help to expand the market.

### 4.4. The Innovation Stage

As the top layer in the digital transformation framework, the innovation stage guides the development direction of technology and business by introducing innovations to both fields. Two innovation principles, partition and integration, are proposed to accelerate innovation taking place in technology and business. Below, we detail how the principles are applied with examples.

#### 4.4.1. Partition in Business

Partition in business simply means to split a part of functions or services from existing products and incubate the one that has a potential to grow into a new business sector.

**Figure 7:**
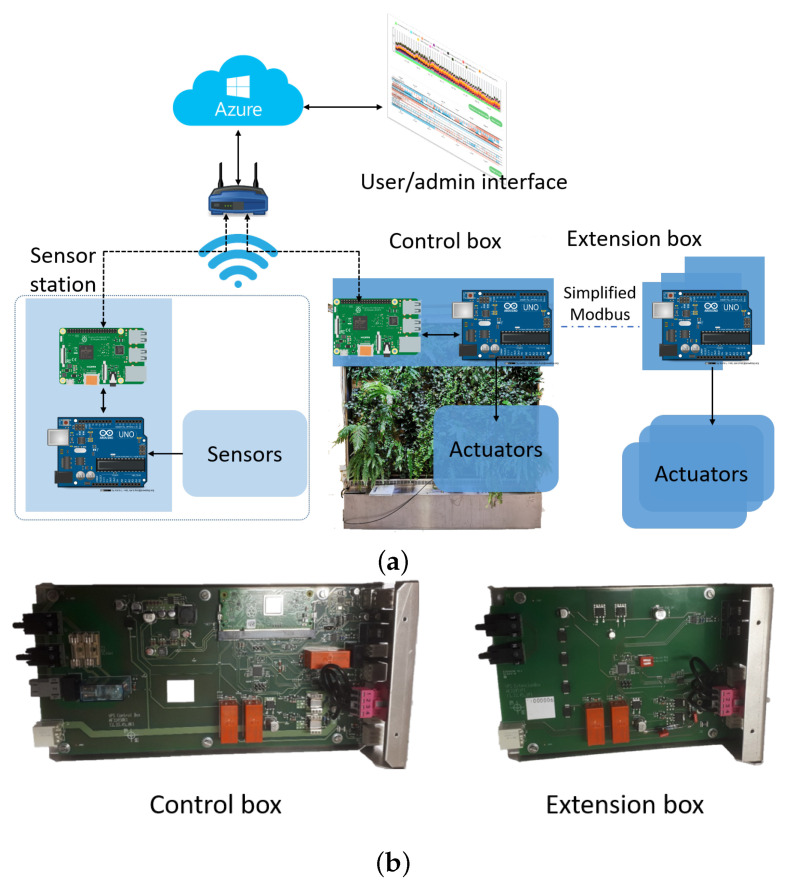
(**a**) The initial digital solution is partitioned into sensor station, control-box, and extension-box. (**b**) The final hardware implementation of control-box and extension-box for vertical plant walls.

In the case of VPS, in light of the great needs for indoor environment monitoring demanded by real estate owners, the remote monitoring feature is split as a separate product and service. As shown in Figure 7a, the initial digital solution for vertical plant walls is partitioned into a sensor station and a control box. The sensor station reserves the sensor portfolio but removes actuators. The remaining components such as Internet access, cloud infrastructures, and the visualization interface, are kept consistent so as to offer a digital service specific to remote indoor climate monitoring. Based on the idea, more value propositions are proposed, e.g., to serve as an additional perceptron to existing building automation systems and to offer specific VOC concentrations according to needs, to name a few. This sensor station has led to another ongoing project, and the business model shall be analyzed by iterating the proposed three-stage procedure in the framework.

#### 4.4.2. Partition in Technology

Partition in the technology implies separating a technical solution into several modules to achieve added benefits. This has been reflected in both software and hardware development. One example is shown in Figure 7a, a partition of the control box is modularized as an extension box that is dedicated to the control of actuators. A simplified Modbus protocol is developed to enable communications. The hardware implementations of the control and extension boxes are shown in Figure 7b. This partition allows VPS to freely increase the width of a plant wall by deploying more extension boxes while lowering the hardware cost.

In the application layer, applications hosted in the cloud platform are encapsulated into several service modules with isolated compute resources assigned, e.g., IoT hub service used for device management and traffic routing, function applications used to insert data, SQL storage services used for storing sensor data, blob storage used for unstructured data, web applications used for human-machine interface, logic applications used for alarm function, and a monitor service used to check application health. Compared to having a monolithic application running in a single virtual server, this modular structure guarantees that a malfunction of one service will have limited interference on other services so as to improve the quality of service.

#### 4.4.3. Integration in Business

In contrast to partition, integrating multiple products or services together may also generate new business values. One potential business development direction for VPS is to provide a platform as a service (PaaS) for stakeholders in similar industries or share the same performance requirements such as indoor farming or smart agriculture. The digital solution that includes hardware, software, cloud infrastructure, and the interface can be offered as a package so as to enable an enhanced business model.

In summary, this section goes through the three-stage digital transformation procedure of VPS. An initial digital solution enabling the four features that are defined by the updated business model is worked out, which consolidates the digital transformation. Further business development can be accomplished by pushing innovation to take place on existing technology and business so as to continuously iterate the three-stage framework.

## 5. Principles in IoT Solution Design

The three-stage framework has provided a guideline for the whole procedure of digital transformation. In addition to the framework, based on the experience of digital transformation in the vertical plant wall industry, several instructive principles for IoT solution development are also summarized for SMEs, aiming to provide assistance for technical selection and consideration.

### 5.1. Time-to-Market First, Prototyping Fast

This principle originates from the agile development method that has been pervasively adopted in the software development process and is equivalently significant for SMEs to develop IoT solutions. It implies that SMEs should not expect a fully polished solution to be achieved before it is delivered to customers but are encouraged to prototype fast and deploy in an early stage. The product and service shall be continuously improved through on-site tests and feedback from users. In the practice of the VPS project, the remote monitoring components for the plant wall were immediately installed on sites once the prototyping was finished, while the other functions such as remote control and visualization interface were still under development. This strategy guarantees that pitfalls in the prototype can be identified at an early time and the solution is verified over a long period.

### 5.2. Cloud First

Cloud computing is the most important enabler to the landing of IoT technologies, benefiting from its high reliability, availability, scalability, and particularly low cost. By taking advantage of cloud computing, SMEs can greatly accelerate the development pace and ease the efforts put into digital transformation. Our previous research result has led to the Culturebee system (http://culturebee.se (accessed on 8 February 2021)), which is a wireless monitoring and control system for Swedish churches and is based on a self-maintained private cloud. However, the solution can hardly be accepted by SMEs such as VPS as the cost is a burden. Therefore, in the plant wall project, a public, cloud-based solution is proposed. Toolsets such as IoT Hub and the IoT software development kit (SDK) offered by Microsoft Azure Cloud can effectively handle trivial functions such as network connection and device management so that we can focus on the implementation of core functions regarding plant walls. With limited monthly cost, compute and storage services in the cloud can be flexibly subscribed and scaled according to needs while eliminating the necessity to maintain hardware infrastructures.

### 5.3. Evolving with New Technologies

Technologies in IoT and cloud computing are rapidly advancing. It is a good practice to keep an eye on emerging technologies and drive the evolution of digital solutions with new technologies. For instance, as cloud computing architecture is evolving towards an edge-cloud computing architecture, and the container technology-based cloud native programming pattern becomes a novel trend in the industry, in the plant wall project, the edge-cloud architecture is benchmarked and put into practice. Based on the new architecture, a part of the cloud functions is offloaded to the edge computing infrastructure that is deployed to the local field to enable capabilities such as data preprocessing and offline storage. Applications are also developed and deployed as isolated container modules to guarantee continuous and large-scale deployment. The details of the edge-cloud architecture application in plant walls can be checked in this study [32].

### 5.4. Asset-Light First

During the development, SMEs should carefully balance the resources spent on the realization of each function block and focus on the implementation of functions tightly related to core value creation. In the practice of VPS, major resources are spent on the implementation of a digital platform that can offer remote sensing and maintenance capabilities, and big, data-enabled smart functions such as anomaly detection and alarm. These asset-light functions will consolidate the business value of VPS after the digital transformation, and thus, are prioritized. Other tasks such as manufacturing, selection of sensors, and hardware component development are either handed over to partners or ordered from market, which maximizes the resources invested in the right direction.

## 6. Discussion

Catering to the digitalization trend, SMEs within the manufacturing industry also envision a digital transformation to take place so as to disrupt the landscape of existing business but are prone to be constrained by their common nature such as being sensitive to cost and their limited expertise in ICT. The framework proposed in this paper aims to provide an applicable guideline for SMEs encountering the digital transformation challenge, as it originates from a real digitalization practice in the vertical plant wall industry. The company VPS has greatly benefited from the proposed framework and the summarized solution design principles for technical selection and consideration in their digital transformation practice. The results include a remote monitoring and management system that is developed based on up-to-date IoT technologies and the Azure public cloud platform. The system enables effective tracking of real-time and historic conditions of indoor environments and plant walls through a uniformed visualization interface. Plant care and maintenance can be performed autonomously and manipulated in a remote manner. A public, cloud-enabled IoT solution also brings considerable improvements in service reliability and scalability while keeping the cost at a low level compared with implementing a private cloud infrastructure. In general, VPS has taken advantage of the framework to achieve digital transformation and started to harvest the fruits of digitalized business.

The three-stage procedure emphasizes a digital transformation that is business-driven, technology-enabled, and innovation-guided. It is not intended to be strictly followed, but more to present a logical chain of how digital solutions are worked out according to business values and how product and business development shall be iterated.

The detailed process of leveraging IoT and cloud computing to realize digital transformation for VPS is presented, as well as several principles that are essential to solution development for SMEs. The solution developed for vertical plant walls can be referenced by companies with similar needs, although it is also under continuous development driven by innovations in technology and business. For example, the cloud computing paradigm is evolving towards edge-cloud computing. Dependency on public cloud platforms can result in vendor lock-in issues and data privacy challenges; thus, a new cloud computing platform that enables services to be deployed across multicloud and hybrid cloud is demanded. In digital transformation practices, these vendor lock-in issues and data privacy challenges can be problematic to SMEs and still do not have a mature solution to address. Therefore, future research on digital transformation frameworks shall cover these topics to provide further guidelines to SMEs.

## 7. Conclusions

Driven by the evolving IoT and cloud computing technologies, in this study, we introduced an applicable framework to SMEs as a reference to perform the digital transformation. A three-stage procedure that covers business, technology, and innovation is proposed to guide SMEs to develop their initial digital solution and iterate it in the long run. A case study of the digital transformation in the vertical plant wall industry is presented in a detailed manner. Several significant design principles that come from practical experience are also presented to accelerate SMEs in the journey of digital transformation. This study can be a start point for SMEs to determine how business can be accelerated by digital technologies and a guideline to pave the way towards digitalization in the industry. As for the future, fine-grained guidelines corresponding to the business, technology, and innovation stages can be researched and proposed to enhance the applicability of the framework. Topics on how to generalize the framework to fit other types of enterprises are also worthy of investigation.

## Data Availability

Data sharing not applicable.
